# Application of Nanomaterials in the Production of Biomolecules in Microalgae: A Review

**DOI:** 10.3390/md21110594

**Published:** 2023-11-16

**Authors:** Xiaolong Yuan, Xiang Gao, Chang Liu, Wensheng Liang, Huidan Xue, Zhengke Li, Haojie Jin

**Affiliations:** 1 School of Food and Biological Engineering, Shaanxi University of Science & Technology, Xi’an 710021, China; xiaolongyuan407@sust.edu.cn (X.Y.); changliu@sust.edu.cn (C.L.); wenshengliang@sust.edu.cn (W.L.); huidanxue@sust.edu.cn (H.X.); zkli@sust.edu.cn (Z.L.); 2 The College of Forestry, Beijing Forestry University, Beijing 100083, China; haojie@bjfu.edu.cn

**Keywords:** nanomaterials, microalgae, metabolites, exopolysaccharides, bioactivity

## Abstract

Nanomaterials (NMs) are becoming more commonly used in microalgal biotechnology to empower the production of algal biomass and valuable metabolites, such as lipids, proteins, and exopolysaccharides. It provides an effective and promising supplement to the existing algal biotechnology. In this review, the potential for NMs to enhance microalgal growth by improving photosynthetic utilization efficiency and removing reactive oxygen species is first summarized. Then, their positive roles in accumulation, bioactivity modification, and extraction of valuable microalgal metabolites are presented. After the application of NMs in microalgae cultivation, the extracted metabolites, particularly exopolysaccharides, contain trace amounts of NM residues, and thus, the impact of these residues on the functional properties of the metabolites is also evaluated. Finally, the methods for removing NM residues from the extracted metabolites are summarized. This review provides insights into the application of nanotechnology for sustainable production of valuable metabolites in microalgae and will contribute useful information for ongoing and future practice.

## 1. Introduction

Microalgae are promising biological factories for the production of diverse natural products, such as proteins, lipids, exopolysaccharides (EPSs), carotenoids, and phenolic compounds [1,2]. These biomolecules or metabolites can be used in industrial products ranging from biofuels, food additives, cosmetics, and pharmaceuticals to alternatives to chemically synthetic or animal-derived products [1,2]. To improve the production efficiency of microalgae-derived metabolites, diverse microalgal cultivation methods have been developed, such as nitrogen deficiency, salinity stress, photoinduction, and carbon source addition [3,4,5,6,7]. In recent years, nanotechnology or nanomaterial application has arisen as a new strategy in the production of valuable metabolites or bioproducts in microalgae.

Nanomaterials (NMs) are materials that are manufactured with at least one dimension being less than 100 nanometers (nm). They have been found to influence the physiology and metabolism of algal cells by generating cell shading, physical damage, and oxidative stress [8,9,10]. Most previous studies emphasized the toxic effects of NMs on microalgal cells [8,9,10]. However, given their nature as nanotechnology, NMs should also have positive roles in regulating cell growth and enhancing metabolite production. Current research progress shows that the concentration and chemical properties of NMs are key for this positive role. For example, the cell biomass of microalga *Chlorella* sp. (Chlorophyta, Trebouxiophyceae) UJ-3 was significantly increased when the cells were treated with low concentrations of Fe_3_O_4_ nanoparticles (NPs), whereas total lipid content was increased at high concentrations of Fe_3_O_4_ NPs [11]. In another study, carbon nanomaterials (CNMs) at a proper concentration could enhance cell growth and EPS production of *Nostoc flagelliforme* (Cyanophyceae) as well as improve the functional activities of the EPS [12]. In addition, some NMs exhibited excellent light-harvesting abilities, which could compensate for the shortcoming of underutilization of light in microalgae cultivation [13,14]. In most cases, the NM concentration is a very important factor that needs to be carefully considered.

The extraction of microalgal metabolites, especially intracellular metabolites, is an important step in the microalgae industry. NMs, having a small size and extremely large surface area, show a strong interaction with cell walls and result in cell wall thinning or disruption, which improves the metabolite harvesting rate [15,16,17]. However, these extracted compounds, such as astaxanthin [18] and EPS [12], were also found to contain the NM traces. These residues may have effects on the functional properties of metabolites or have health risks. Several technologies for removing them from the harvested metabolites have been developed.

Nanotechnology is a relatively young but rapidly developing field. The information on the positive application roles of NMs in the microalgae field is relatively fragmented. In this review, we aim to provide a systematic summary of the application of NMs in microalgae cultivation and metabolite production, as well as their effects on the extraction and functional modification of metabolites and their removal technologies from the metabolite products. In addition, the underlying mechanisms are also summed up, which are largely dependent on the small size, particle concentration, and chemical properties of NMs. Cell growth is basal for biomass increase and metabolite harvesting. Thus, the rapid development of diverse NMs applied to enhancing photosynthetic utilization efficiency is particularly focused on in this review. Despite some positive progress, there is still a gap from laboratory research to industrial application. We believe that this review will contribute useful information for ongoing and future practice in the microalgae industry.

## 2. The Effects of NMs on Microalgae Growth

### 2.1. The Interaction of NMs and Microalgal Cells

How NMs exert effects on cell growth is an important issue. They may be distributed around cells or penetrate the cell wall to function. Several reviews have summarized the interactions of NMs and microalgal cells, including adsorption, distribution, and ecotoxicity [19,20,21]. Several examples show their contact relationships. NMs can be distributed around cell walls when applied in microalgae cultivation, which limits cell exposure to nutrients and light [22]. Those NMs with a size smaller than that of the cell wall pore were supposed to enter the cells directly [23]. Larger NMs could penetrate the cell wall through embedding [24], membrane perforation, and endocytosis [21]. After entering the cell, NMs could physically contact various organelles and damage or alter their structures and functions [25,26]. Thus, it is critical to examine the mode of interaction (extracellular or intracellular) when discussing the effects of functional mechanisms of NMs applied in microalgal cultivation.

### 2.2. Improving Photosynthetic Utilization Efficiency

**Figure 1:**
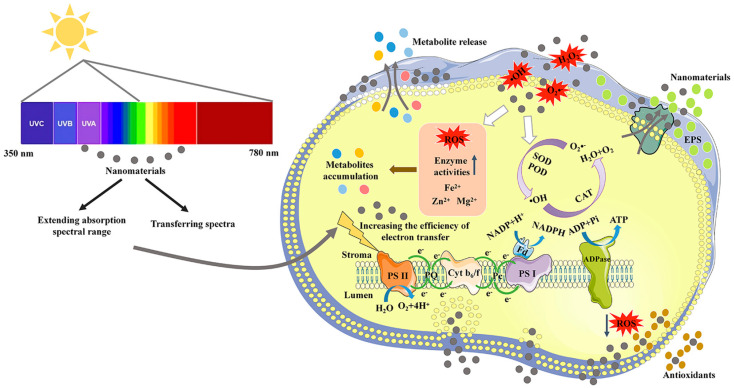
A mechanistic illustration of the application of nanomaterials in improving photosynthetic utilization efficiency and metabolite production of microalgae.

As the main form of energy used by algae, light is one of the most important factors affecting microalgal growth. Light energy is absorbed and transmitted through photosynthetic pigments, including chlorophyll, carotenoids, and phycobiliprotein, in photosynthetic systems. However, these pigments can only cover no more than 10% white light [27]. As the main photosynthetic pigments, chlorophyll a and b have only a dual-absorbance range of blue (450–480 nm) and red (605–700 nm) lights [27]. To maximize the use of solar energy, developing high-performance light conversion materials to improve the absorption efficiency of red and blue lights or utilizing the lights of other wavelengths for growth may be a feasible approach [28,29,30]. A mechanistic illustration of the application of NMs in improving light utilization efficiency and growth of microalgae is shown in Figure 1.

#### 2.2.1. Increasing the Absorption of Red and Blue Lights

In liquid suspension cultivation, cell shading can cause insufficient absorption of blue and red lights by chlorophyll molecules [31]. Light utilization efficiency can be improved by light-harvesting NMs to maintain normal photosynthetic systems in microalgae [32]. One of the mechanisms is to enhance red or blue light absorption of microalgal cells. It was reported that some NPs selectively enhanced microalgal blue light absorption through plasma light scattering, increasing the growth of *Chlamydomonas reinhardtii* (Chlorophyta, Chlorophyceae) and *Cyanothece* (Cyanophyceae) 51142 by more than 30% when exposed to the full spectrum [33]. Additionally, the localized surface plasmon resonance (LSPR) of metal NPs was used to filter light at specific wavelengths within a bioreactor, and the LSPR wavelength could be tuned to the violet-blue or red regions [34]. The photosynthesis of Mung Bean was increased by more than two-fold, and the dry weight was increased by 15.39% [34].

#### 2.2.2. Spectral Transformation of Infrared Light

Near-infrared (NIR) light, accounting for approximately 52% of the solar spectrum, is not effectively utilized by photosynthesis [35,36]. The photosynthetic efficiency will be improved if NIR light can be utilized by microalgae. A feasible route is to convert NIR light into visible light with photon-up conversion (UC) materials [37]. It was reported that NaYF_4_:Yb,Er, as a UC material, could efficiently transform NIR light to visible light (mainly green and red lights) via multiple-photon absorption [38,39]. Due to the potential of carbon dots (CDs) in light conversion, some studies explored the joint effect of NaYF_4_:Yb,Er, and CDs. For example, the construction of NaYF_4_:Yb,Er + CD nanocomposites further improved the conversion of NIR light to red light [40]. CDs modulated the UC emission of NaYF_4_:Yb,Er by efficient energy transfer [40].

#### 2.2.3. Spectral Transformation of Yellow and Green Lights

In the photosynthetic system, the wavelengths at 500–600 nm (yellow and green lights) are not well absorbed by microalgae [27]. To improve the absorption efficiency of these lights, light-trapping NMs can be developed to convert poorly absorbed yellow and green lights into highly absorbed visible lights. Among light-trapping NMs, CDs were applied as good electron donors or acceptors in light energy conversion [41,42,43,44] due to their high quantum yield, chemical stability, and superior biocompatibility [45]. At present, these CDs can emit red light under yellow-green or green light. For example, the CDs with tunable emission could directionally shift unutilized yellow-green light (500–600 nm) to red light (580–700) to promote growth by 15% in *Chlorella* sp. [13]. They could also enhance microalgal photosynthetic activity by redshifting the incident light [46]. However, the synthesized CDs did not emit blue fluorescence under yellow or green light [46]. Under 1 mg/L CDs treatment, the photosystem II activity of *Chlorella* was significantly enhanced, and the growth rate was increased by 52.7% [46]. In addition, artificially synthesized polyolefin-based fluorescent dyes could convert green light (500–570 nm) to red light (580–650 nm) and contribute to microalgal biomass increase [14].

#### 2.2.4. Spectral Transformation of UV Light

Ultraviolet (UV) light (200–400 nm) usually has negative effects on microalgal growth and is also poorly adsorbed by microalgal photosynthetic systems [47]. In microalgae, it has rarely been reported that light-trapping NMs can convert UV light into highly absorbed visible light [48]. However, two aggregation-induced emission luminogens (AIEgens) were recently reported to absorb UV/blue light to emit green and yellow lights, which were efficiently used by *Cyanobium bacillare* (Cyanophyceae) [49]. Under AIEgens treatment, several photosynthetic parameters were significantly improved, and the growth (in terms of cell density) of *C. bacillare* was boosted five-fold [49]. More reports about UV light conversion are provided in the plant research area. For example, the synthesized CDs were reported to emit blue fluorescence under UV excitation [43,50,51]. Functionally modified CDs could enhance the spectral transformation of UV light, such as increasing graphitic-N and hydroxyl group contents [42], vinyl alcohol encapsulation [52], and amine functionalization [52]. A multifunctional CD was reported to match the chloroplast absorption spectrum (blue and red lights) by strong absorption of UV light [53]. In addition, aggregation-induced emission carbon dots (CD-AIEgens) had strong UV absorption in natural light [54], exhibiting application potential in mass cultivation.

#### 2.2.5. The Quenching Effect and Stability Maintenance of Light Harvesting NMs

The aggregate state of NMs may cause the quenching effect of fluorophores in water, which could decrease the efficiency of spectral conversion. To solve this problem, sustainable aggregation-induced emission (AIE) materials were prepared from natural resources [55,56,57]. These resources include quercetin, lignin, and rosin, which can eliminate the quenching effect of the existing materials developed to enhance photosynthesis [56,57,58]. In the state of aggregation, AIE materials produced stronger excitation than traditional aggregation-caused quenching luminescent NMs [59,60]. In addition, the coupled application of AIEgens and CDs could obtain unique optical properties, such as high AIE-active fluorescence, efficient harvesting of UV light, and good photostability [55].

To avoid the aggregation-caused quenching in light-harvesting NMs, special scaffolds have also been developed, including macrocycles [61,62,63,64], DNA [65,66], and cyclic peptides [67,68]. The design and synthesis of ideal scaffolds are complex. To greatly simplify the fabrication steps of special scaffolds, the employment of AIE materials was presented [69,70]. In addition, supramolecular polymerization is an excellent strategy for constructing light-harvesting NMs, which can assemble the chromophores together to pack tightly and enhance supramolecular assembly-induced emission of the chromophores [70]. New polymerizations have been reported based on ureidopyrimidinone quadruple hydrogen bonding units [71] and tetraphenyl-ethylene [72]. Organic dyes are classical optical materials that can be used in AIE NPs. A hybrid dye system based on the tetraphenylene-encapsulated organic dye (Nile red) was synthesized, which had a considerable redshift distance (~126 nm), with a high energy-transfer efficiency of 99.37% and an antenna effect of 26.23% [73].

The NM solutions are easily dried or washed away when applied to plants and algae [74]. To gain a prolonged enhancement of photosynthesis by NMs, continuous leaf spraying or hydroponic conditions throughout the plant growth process are needed, but this results in high labor costs or large-scale facility construction [41,75,76]. In vitro spraying of adhesive fluorescent coatings on leaf surfaces with continuous fluorescence emission and good rain–erosion resistance provides a new tool for efficient photosynthesis enhancement [74]. CDs and some monomers could be used to synthesize covalently cross-linked polymers with prolonged fluorescent capacity, rain–erosion resistance, and stability [77]. In addition, a fluorescent polymer coating was developed, which consisted of UV-excited, blue light-emitting nitrogen-doped CDs as the fluorescent body to catalyze the covalent copolymerization of CDs and tannic acid [77]. This fluorescent polymer coating exhibited excellent fluorescence properties, stability, nontoxicity, and rain–erosion resistance [77]. Such light harvesting NMs used in plants should also have good potential to be applied in microalgae cultivation for enhancing photosynthetic efficiency.

### 2.3. Removing Reactive Oxygen Species

Various environmental stressors can induce reactive oxygen species (ROS) in microalgal cells, generating oxidative stress [47]. Intracellular antioxidants from algal cells can mitigate cellular damage. However, the effectiveness of these antioxidants is sometimes limited. Recently, antioxidant NPs were synthesized by amalgamation of material sciences with nanotechnology, including carbon nanotubes (CNTs), metal NPs, and metal oxide NPs [78,79]. In addition, the transport function of NMs was developed to transport antioxidants into cells to oxidative stress damage [80].

#### 2.3.1. The Antioxidant Activities of NMs

Some NMs can scavenge ROS and mimic antioxidant molecules. Pristine CDs reduced the UV light damage by scavenging 2,2-diphenyl-1-picrylhydrazyl (DPPH) radical in *Chlorella vulgaris* (Chlorophyta, Trebouxiophyceae) [81]. The antioxidant activity is related to the long-conjugated C=C chains of the CDs [82]. Similarly, cerium oxide NPs (CON) exhibited superoxide dismutase (SOD) activity, which reduces the levels of superoxide anions [83,84]. The antioxidant level of CON is related to nanocrystal diameter since smaller diameter nanocrystals are found to be more reactive towards H_2_O_2_ [85]. CON could be applied multiple times and used for several weeks since the particles remained colloidally stable [85]. To further improve the ROS scavenging ability under broad-scale environmental conditions (e.g., application in biological tissues and cells), CON could be wrapped with biocompatible polymers, such as PEGylated and dextran [86,87]. The dextran-coated CON was applied in the chloroplasts of photosynthetic organisms to improve the antioxidant capacity of their photosynthetic system [35,88].

Functionalized NMs derived from various biological extracts of living organisms, such as proteins, EPSs, and terpenes, show potential antioxidant activity. For example, extracellular protein (from *Escherichia coli*)-capped gold NPs showed excellent antioxidant ability [89]. The DPPH radical scavenging activity of gold NPs was found to be dose-dependent, with the maximum inhibition being greater than that of the extract alone [89]. The EPS-mediated silver (Ag) NPs also showed excellent antioxidant activities at a suitable concentration [90]. Terpene-rich extracts were used to synthesize the antioxidants of Ag NPs [77]. The obtained antioxidant ability was comparable to that of ascorbic acid [91]. In addition, the crude extracts of living organisms were also used to synthesize NM antioxidants, including leaf extracts [92,93,94], cell-free supernatants [94,95], and other extracts [96,97,98].

#### 2.3.2. The NMs-Facilitated Transfer of Antioxidants

Many chemical compounds, either endogenous or exogenous, have been evaluated for their antioxidant properties, which have the potential to modulate oxidative stress [99]. Recently, NPs were found to efficiently enhance antioxidant activity and provide targeted delivery of certain antioxidants that show poor cell membrane permeation and cell internalization [80]. Encapsulation of vitamin E and catechol in Ag NPs facilitated the scavenging of DPPH radical, hydrogen peroxide, and nitric oxide [100]. The Ag NPs were also therapeutically applied for targeted delivery in breast cancer treatment [100]. Biodegradable polyanhydride NPs containing the mitochondrion-targeted antioxidant apocynin were explored for treating neuronal cells, and their pretreatment significantly protected cells against H_2_O_2_-induced toxicity [101]. The curcumin-encapsulated NPs with dual responses to oxidative stress and reduced pH could efficiently reduce the excess oxidants produced by lipopolysaccharide-stimulated macrophages [102]. These studies provide useful references for decreasing the damage of oxidative stress in microalgal cells generated from adverse environmental conditions. Moreover, precise delivery by NMs to specific locations, such as cell membrane, chloroplast, and nucleus in microalgae, needs to be explored with the aim of advancing the development of microalgal biotechnology.

## 3. The Effects of NMs on Metabolite Production

In addition to the positive impacts of NMs on photosynthesis and growth, their potential to promote metabolite accumulation has also attracted increasing attention in recent years. These metabolites include lipid, carotene, astaxanthin, phenolic compounds, and EPS.

### 3.1. Lipid Production

Microalgal lipids are called third-generation bioenergy because the production process does not alter the food chain and results in less pressure on arable lands and the environment [103]. Currently, the techniques for producing microalgal lipids include strain selection, optimization of cultivation conditions, photobioreactor design, and lipid harvesting. Among them, optimization of the cultivation conditions, such as abiotic stress treatment, is an efficient tool to improve lipid production. Recently, the unique properties of metallic NPs in promoting lipid accumulation have attracted much attention [104,105,106].

The chemical composition of NPs is an important factor affecting lipid productivity. For example, SiC and g-C_3_N_4_ NPs improved biomass and lipid accumulation, while TiO_2_ and TiC NPs had an inhibitory effect on biomass increase [107]. Fe or Mg NPs exhibited remarkable abilities in improving the lipid content due to their release of disassociated ions (Fe^2+^ or Mg^2+^), which are essential to photosynthetic electron transport and chlorophyll synthesis [108]. Under 100 mg/L nano Fe_2_O_3_, total lipid content in *Scenedesmus obliquus* (Chlorophyceae) was increased by 44.8% compared to non-treated control, while total lipid content was increased by 53.4% under 100 mg/L nano MgO [108]. The effectiveness of lipid production is associated with the concentration of nano Fe_2_O_3_ or MgO. Properly elevated Fe concentration (0.65~6.5 mg/L) could promote lipid production in *Chlorella sorokiniana* (Chlorophyceae) [109], while excessive Mg concentration (1 g/L) from MgSO_4_ NPs could also enhance lipid production in *C. vulgaris* [110]. In addition, different types of Fe NPs showed different effects on lipid production [11,108,111]. After Fe_3_O_4_ NP treatment, total lipid production was increased by 71.7% compared to the normal culture in *Chlorella* sp. UJ-3 [11]. After α-Fe_2_O_3_ NP treatment, the total lipid yield was increased by 44.8% in *Tetradesmus obliquus* (formerly *Scenedesmus obliquus*) (Chlorophyceae) [108]. After zero-valent iron NP treatment, the total lipid yield reached 8.72 g/L in *Parachlorella kessleri* (Chlorophyta, Trebouxiophyceae), being the highest production among the tested algal strains [111].

The concentration of NPs can affect neutral lipid and total lipid accumulation. The neutral lipid fluorescence intensity of *T. obliquus* was significantly promoted by increasing the concentration (within 100 mg/L) of CNTs, nano MgO, and nano Fe_2_O_3_ [108]. The total lipid content in *Chlorella* sp. UJ-3 was not significantly changed at a low concentration (within 50 mg/L) of Fe_3_O_4_ NPs, while at a high concentration (50–200 mg/L), it was significantly increased [11]. Thus, total lipid production was increased, but algal biomass was decreased at the high concentration of Fe_3_O_4_ NPs [11]. Actually, low concentrations of NPs usually stimulate algal photo-physiological activity and growth, while high concentrations of NPs inhibit cell growth [12,112]. A two-step strategy is often adopted to resolve the contradiction between biomass increase and lipid production: firstly, algal cells are exposed to low concentrations of NPs to increase biomass; secondly, algal cells are treated with high concentrations of NPs to stimulate lipid accumulation. Notably, the proper incubation time in the first phase is sometimes dependent on the NP property. When *C. vulgaris* was treated with four metal NPs, visible growth was observed after 8 days in the presence of copper NPs, 13 days in magnesium NPs, 7 days in lead NPs, and 11 days in zinc NPs [113].

The capability of single NP treatment in promoting lipid production is usually limited, while a combined treatment often leads to higher production. Under xenon lamp illumination, microalgal growth and lipid accumulation were enhanced by the addition of SiC NPs [108]. The total lipid content reached 40.26% at 150 mg/L SiC NP with a photoperiod of 6:18 h [108]. The addition of N-CDs, which exhibit strong blue emission with bright luminescence, promoted lipid accumulation with an increase of 37.96% in magnesium amino-clay (MgAC)-containing culture medium [114].

### 3.2. Carotenoid Production

Carotenoids are natural tetraterpenoids that can be produced by microalgae. They are widely used as nutraceuticals and natural colorants in the cosmetic and food industries and are also applied in chemotaxonomy and therapeutics [115]. Technologies for producing carotenoids, such as abiotic stress treatment (e.g., high salinity and strong light), have also been developed [116]. As novel stressors or modulators, metals, metalloids, and metallic NPs can also affect microalgal metabolism and carotenoid production [117,118].

#### 3.2.1. β-Carotene Production

*Dunaliella salina* (Chlorophyceae) is considered the best commercial source of β-carotene [115]. The application of NMs in *D. salina* cultivation may enhance β-carotene production. Recently, Bi_2_O_3_ and Gd_2_O_3_ NPs treatments were found to increase carotenoid production in *D. salina* compared to the untreated culture [116]. In addition, a two-step cultivation method was adopted to improve both biomass and carotenoid yields in *D. salina* under MoS_2_ NPs exposure [118]. In step one, under 50 µg/L MoS_2_ NP exposure, biomass production was increased by 1.33 folds compared to the control; in step two, followed by 30 days of normal light cultivation, MoS_2_ NP-treated cells were subjected to high light treatment for 7 days, which increased the β-carotene content by 1.48 folds [118]. It suggests a promising prospect for the combined application of NMs with environmentally stressful conditions. Regulating the light wavelength by NMs is also a potential method to promote carotenoid production. Eroglu et al. placed spheroidal Ag NPs and gold nanorods around *C. vulgaris* culture flasks, backscattering in the spectral regions favorable for cell growth, resulting in the increased biosynthesis of carotenoids [119].

#### 3.2.2. Astaxanthin Production

Astaxanthin is a red secondary ketocarotenoid pigment and has strong antioxidant activity [120]. *Haematococcus lacustris* (Chlorophyceae) is considered one of the best sources of astaxanthin (~4% of dry weight) globally [121,122]. Stressful conditions have been reported to induce astaxanthin production, such as high salinity, nutrient deficiency, strong light irradiance, and high temperature [123,124,125]. The application of nanobiotechnology should be beneficial for further enhancing the astaxanthin production capability in *H. lacustris*. For example, magnesium NPs were proven to enhance astaxanthin production in *H. lacustris* [18]. Besides magnesium, Zn plays structural, catalytic, and co-catalytic roles in over 300 enzymes, which are involved in nucleic acid metabolism and protein synthesis [126]. Fe is necessary for numerous enzymatic reactions and photosynthetic electron transport chains [127,128]. Therefore, the NPs synthesized with biologically essential elements (Zn or Fe) had a positive effect on astaxanthin accumulation [129]. The combined application of Zn NPs and Fe NPs could further improve the accumulation of astaxanthin [129].

The average particle size of NPs also affects astaxanthin production. Nasri et al. synthesized ZnO NPs by sodium hydroxide (chemical method) or pomegranate peel extract (green method) as reducing agents [18]. The average particle size of the former was approximately 80 nm, and that of the latter was approximately 30 nm [18]. Treatment with the latter resulted in a relatively high astaxanthin content in the dry biomass of *H. lacustris* [18].

MgAC NPs could induce the biosynthesis of astaxanthin in *H. lacustris* due to elevated intracellular ROS, and the astaxanthin production reached 302 ± 69 pg/cell in the presence of 1.0 g/L MgAC NPs, being 13.7 folds than that in the untreated control (22 ± 2 pg/cell) [130,131]. In addition, the amine components in MgAC NPs could accelerate CO_2_ absorption and thus also promote algal growth [132].

It was reported that light at a specific wavelength, such as blue light, could efficiently increase the productivity of astaxanthin in *H. lacustris* [133,134]. Light-harvesting CNMs can regulate certain wavelengths to realize this strategy at a low cost. Abu-Ghosh et al. synthesized nontoxic nitrogen-doped carbon dots (N@CDs) with high fluorescence quantum yield, and N@CD treatment (1 mg/L) improved astaxanthin production more than two folds in *H. lacustris* compared to the untreated control [135]. However, the photostability and dispersibility of N@CDs limit their application in large-scale biological processes. A simple and eco-friendly approach for large-scale synthesis of liquid-type fluorescent carbon nanodots (C-paints) was developed [121]. The C-paints exhibited a carbonyl-rich surface with excellent photostability, fluorescence efficiency, and good biocompatibility [121]. The C-paints at a concentration of 1–10.0 mg/mL could induce an approximately >1.8 times higher astaxanthin content than that in the control cell [121].

### 3.3. Phenolic Compounds and Protein Production

Phenolic compounds, which have shown diverse bioactivities, are natural molecules that are found mainly in plant tissues and microalgae [136,137,138]. AgNPs could enhance phenolic compound production and improve their biological activities (antioxidant, antimicrobial, and anticancer) in genetically transformed root cultures of *Cucumis anguria* (Magnoliophyta) [137]. In microalgae, NPs have also been reported to exert effects on phenolic compound production [138]. In particular, 15% N-doped anatase resulted in high production of phenolic compounds, reaching 65.2 and 68.0 mg/g for *H. lacustris* and *Arthrospira platensis* (Cyanophyceae), respectively [138].

At present, there are relatively few research studies studying the impact of NMs on protein accumulation in algae. One study showed a negative effect of single-wall carbon nanotubes (SWCNTs) on protein accumulation in *Microcystis aeruginosa* (Cyanophyceae) [112]. However, several studies have reported positive effects. When exposed to nano ZnCl_2_ (within 32 μM), the protein content could reach 130.8 μg/mL in *Desmonostoc muscorum* (Cyanophyceae) [139]. When exposed to 90–720 μg/L AgNPs, the protein content was significantly increased by 110–293% in *C. vulgaris* compared to the control, and it even could reach a maximum of 583% increase at 1440 μg/L AgNPs [140]. The effects of differently oxidized graphene oxides (GOs) on the protein content of *Raphidocelis subcapitata* (Chlorophyceae) and *Synechococcus elongatus* (Cyanophyceae) were compared [10]. Three GOs were found to increase the extracellular protein production of both algal strains [10]. Expanded application of NMs in promoting protein production in microalgae is still expected in future research.

### 3.4. EPS Production

In recent years, microalgal EPSs have attracted increasing attention due to their beneficial bioactivities [141,142]. Developing new strategies is helpful to further improve EPS production efficiency. The regulation of culture conditions has been widely adopted to promote EPS production [143,144]. As the exogenous additive, NMs could also stimulate the secretion of extracellular polymeric substances (consisting of EPS and protein) of microalgae [145,146]. The latest study reported the potential effect of four CNMs, including graphene, GO, multiwalled carbon nanotubes (MWCNTs), and aminated multiwalled carbon nanotubes (MWCNT-NH_2_), on EPS production [12]. Under 15 mg/L GO treatment, the biomass was improved by 11.1%, and the EPS production was increased by 36.1% compared to the non-treated control [12].

### 3.5. The Mechanisms of NMs-Facilitated Metabolite Biosynthesis

The potential mechanisms of NMs-facilitated metabolite production in microalgae are described in Figure 1, including the promotion of photosynthetic utilization efficiency and regulation of ROS levels. The related NMs, microalgae and metabolites are summarized in Table 1. Photosynthetic utilization efficiency in microalgae is improved by extending the absorption spectral range and spectral transformation of poorly absorbed light. Improving photosynthetic utilization efficiency can increase both biomass and metabolite production.

The levels of ROS, including hydrogen peroxide (H_2_O_2_), hydroxyl radicals (^•^OH), and superoxide radicals (O_2_^•−^), are of great significance to maintaining the stability of the cellular redox environment. A high level of ROS can be detrimental to cellular homeostasis and exert toxic effects on cells. A moderate increase in ROS levels can trigger defense responses and thus stimulate cells to synthesize reductive biochemicals or secondary metabolites. For this reason, various ROS-based stress conditions, such as intense light, salinity stress, nitrogen restriction, and high temperature, are employed to stimulate the biosynthesis of target metabolites. NMs show promising potential in enhancing metabolite production by causing oxidative stress in microalgae in two ways. In the first way, NMs at a suitable concentration can create an oxidative stress environment and stimulate cells to synthesize antioxidative biochemicals, such as lipids, astaxanthin, and EPS. The second way is indirect. NMs have numerous active sites on their surface, and these reactive sites can act as electron donors or acceptors that react with molecular O_2_ to form ROS [147]. The capability of NMs to generate ROS is dependent on their size, surface area, surface chemistry, and concentration [131].

NMs may stimulate the activity of some enzymes and thus affect metabolite biosynthesis. ACCase is a rate-limiting enzyme that catalyzes the first step in fatty acid biosynthesis [148]. One study showed that NMs could increase ACCase activity and result in increased lipid production [107]. The mechanisms of EPS accumulation after NM exposure also have some clues. In addition to the aforementioned ROS stimulation, one study reported that elevated Ca^2+^ levels stimulated the production of extracellular polymeric substances (mainly EPS and protein) [149]. In this study, the Ca^2+^ levels were improved by 50–300% under SiO_2_ NM treatment [149]. The EPS biosynthesis comprises several steps: synthesis of sugar nucleotides, assembly of the repeating units, polymerization of the repeating units, and export of the polymer to the cell surface [150,151]. It was reported that glycosyltransferase, an enzyme for assembly of the EPS repeating units, was potentially critical for EPS synthesis in *N. flagelliforme* [152]. Exploration of the mechanisms associated with NMs-induced EPS biosynthesis should pay more attention to those polysaccharide biosynthesis-related enzymes, including glycosyltransferases.

## 4. The Effects of NMs on Metabolite Extraction

After microalgae cultivation, their metabolites need to be extracted. Some pretreatment methods have been used for microalgal cell disruption, such as physical treatment (ultrasonication, thermal, microwave, bead milling, and cryogrinding), chemical methods (acid-alkaline, solvent soaking, and osmotic shock), and enzymatic treatment [17]. Nanotechnology provides an alternative or supplementary approach to cell disruption. Due to the tiny structure of NPs, pretreatment with NPs can rapidly lyse the cell wall to release intracellular metabolites [153]. For example, Ag NPs were used to lyse microalgal cell walls for lipid and carbohydrate extraction [17]. The oil extraction yield was increased by 8.44–17.68% when treated with Ag NMs at a concentration range of 50–150 µg/g [154]. The mechanism of cell lysis by NMs is related to their strong contact with the cell wall, which leads to the formation of ‘‘pits/holes’’ in cell walls [12,155,156].

Researchers have also developed multifunctional NMs to facilitate algal cell harvesting and cell wall breaking [157]. For example, cetyltrimethylammonium bromide decorated Fe_3_O_4_ NPs could exert both flocculant and cell disruption functions [158]. This material enabled the effective harvesting of 96.6% *Chlorella* sp. KR-1 at a dosage of 0.46 g particle/g cell [158]. The use of Fe_3_O_4_-TiO_2_ NPs harboring chitosan-coated core-shell structures could obtain a >98% harvest rate of *Mychonastes homosphaera* (Chlorophyceae) and reach a 96–97% recovery rate of intracellular lutein and lipid [159]. To reduce energy consumption, ZnFe_2_O_4_ octahedrons were constructed using a hydrothermal method and then functionalized with amino silane [160]. The resulting ZnFe_2_O_4_ magnetic flocculant enhanced algal cell wall lysis by affecting the photocatalytic Fenton reaction under simulated sunlight irradiation with the assistance of H_2_O_2_ solvent [160]. In addition, trifunctional carbon NPs filled with Fe_3_O_4_ were synthesized and applied in microalgal harvesting and lipid extraction and entrapment [161].

## 5. The Effects of NMs on the Physiochemical Properties of EPS

Microalgal metabolites, particularly EPS, have a wide range of bioactivities [162,163]. The functional activities of EPSs are generally determined by their structural features [164]. To date, the changes in EPS activities upon NM treatments have been relatively less reported. Some progress is summarized in this section.

### 5.1. The Structural Modification of EPS

After Ag NMs treatment, Ag NMs-EPS binding was visualized, in which Ag NMs were trapped by a fibrillar network of the polysaccharide secreted from *Cylindrotheca closterium* (Bacillariophyceae) [165]. EPS assembly could also be accelerated to generate microgels [166], which suggests the possibility of the structural modification of NMs on EPS. Ag NPs could weaken the spectral intensity of the peak that was α-linked in the glycosidic linkage of aldopyranose, indicating that the functional group of EPS was damaged [167]. Similarly, the aldehyde group of polysaccharides was oxidized to a carboxyl group under Ag NPs exposure [168]. Recently, Yuan et al. reported that the monosaccharide compositions of EPSs were obviously altered by four CNM treatments, and the functional groups of the CNM treatment-resulting EPSs were also changed [12]. A new peak, representing C–H out-of-plane flexural vibration, occurred for the G or GO treatment-resulting EPS; the MWCNT-NH_2_ treatment-resulting EPS lacked the peak of the pyrenoid structure; and the MWCNT treatment-resulting EPS lacked the peak of β-type glycosidic linkage [12]. All these results provide definitive evidence showing the structural modification of EPS by NMs.

### 5.2. The Functional Modification of EPS

NMs may also exert effects on the functional alteration of EPS during microalgae cultivation, except for culture conditions [141,169]. CNMs at a suitable concentration were reported to alter the functional properties of EPS, including the changes in apparent viscosities, flocculating activities, water absorption and water-holding abilities, and antioxidant activities [12]. For example, the flocculating activity was increased by 33% in the MWCNT-NH_2_ treatment-resulting EPS; the scavenging abilities of the GO treatment-resulting EPS for ·OH radical and DPPH radical were increased by 33% and 58%, respectively. Therefore, CNM treatments have great potential for generating functionally ameliorated EPSs during microalgae cultivation. Of course, if the underlying mechanism is related to elevated intracellular ROS levels, it should also be potentially effective in the functional modification of other metabolites. This is worth exploring in future studies.

## 6. The NM Residues in EPS and Removal

When NMs are applied in microalgal metabolite accumulation and harvesting in microalgae, the extracted metabolites may contain trace amounts of residual NMs. It was reported that when ZnO NPs were used to induce the biosynthesis of astaxanthin in *H. lacustris*, the extracted astaxanthin contained traces of ZnO NPs [18]. Yuan et al. also found NM residues in the CNM treatment-resulting EPSs by scanning electron microscopy [12]. In this section, the effects of trace NM residues on the functions of EPS are discussed, and the methods for their removal are summarized.

### 6.1. The Effects of NM Residues on the Functions of EPS

**Figure 2:**
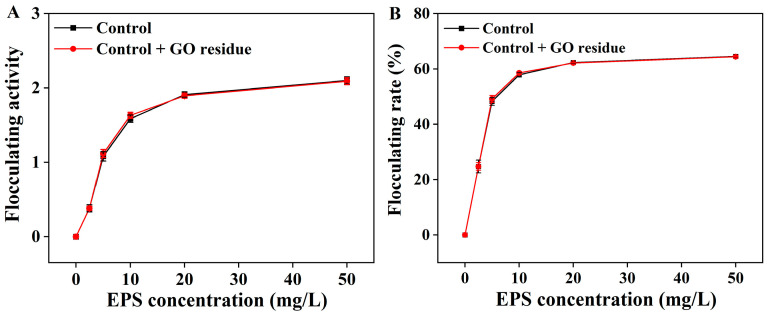
The flocculation activity (**A**) and flocculation rate (**B**) of the extracted EPS with or without trace GO residue. Data shown are the mean ± SD (*n* = 3). The amounts of EPS and GO residue for testing were designed according to reference [12].

**Figure 3:**
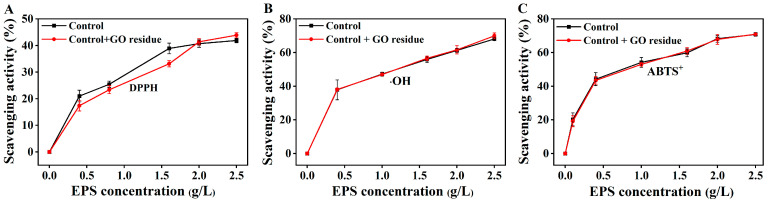
The scavenging abilities of the extracted EPSs with or without trace GO residue for OH (**A**), DPPH (**B**), and ABTS^+^ radicals (**C**). Data shown are the mean ± SD (*n* = 3). The amounts of EPS and GO residue for testing were designed according to reference [12].

It was rarely reported whether trace NM residues could alter the functional properties of EPS. We previously explored the effects of trace CNM residues on the functional properties of EPS (unpublished). As shown in Figure 2, the flocculation activities were not changed in the EPS containing trace GO residue; as shown in Figure 3, the GO residue did not affect the antioxidant abilities of EPS. However, the effect of NM residues on the bioactivities of other metabolites remains unclear.

### 6.2. The Methods for Removing NM Residues

**Figure 4:**
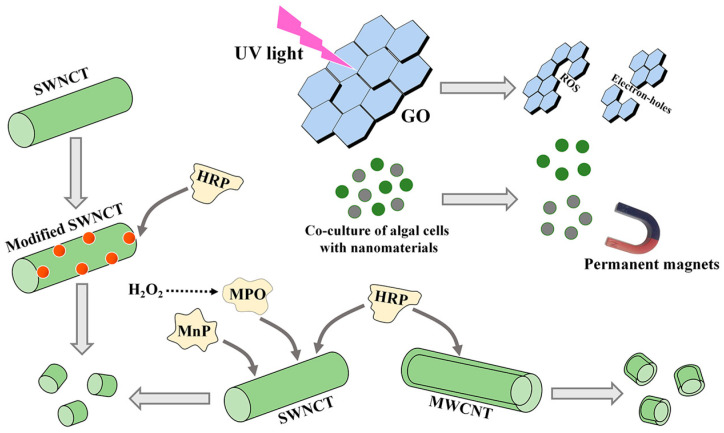
The methods for removing CNM residues from microalgal bioproducts. GO, oxidized graphene; SWCNT, single-walled carbon nanotube; MWCNT, multiwalled carbon nanotube. MPO, myeloperoxidase; MnP, manganese peroxidase; HRP, horseradish peroxidase.

After the treatment of microalgae by NMs, the extracted metabolites, particularly EPS, usually contain trace NM residues. Therefore, it is necessary to develop removal methods for these residues. In this section, three removal methods, including photodegradation, permanent magnet removal, and enzymatic degradation, are summarized (Figure 4).

#### 6.2.1. The Photodegradation and Permanent Magnet Removal

Light, especially UV light, can induce the production of ROS and electron holes, resulting in the degradation and transformation of NMs [170]. In the degradation progress, CNMs were converted into small molecules by generating CO_2_ [170]. The removal efficiency of GO was mostly determined by the quantity of ROS [171]. On the premise of lighting, additional chemicals, such as the simultaneous addition of H_2_O_2_, could increase the degradation rate of CNMs [171]. In addition, the pH and size of NMs also affected the photodegradation efficiency [172].

Magnetic NPs (MNPs) are considered a promising novel harvesting material to obtain algal cells [157]. After algal cell collection, MNPs are separated in flocs by permanent paramagnetic movement. Permanent magnet removal seems to be meaningful for separation due to its simple operation, fast separation, and energy savings. However, these NMs must be magnetic in the co-cultures of NMs and algal cells.

#### 6.2.2. The Enzymatic Degradation

Some enzymes, such as myeloperoxidase (MPO), manganese peroxidase (MnP), and horseradish peroxidase (HRP), can catalyze the biodegradation of CNTs [172]. MPO and MnP mainly degrade SWCNTs, and HRP degrades SWCNTs and MWCNTs [172]. Among these three enzymes, MPO exhibited an excellent ability to degrade SWCNTs in vivo or in vitro, which was related to the produced hypochlorite [173]. The degradation times of the three enzymes for the same NMs are different. When only H_2_O_2_ was used as an auxiliary reactant, MPO could degrade SWCNTs into small molecule products [174].

Additionally, the removal effects of these enzymes for NM residues are also affected by the structures of NMs. For example, it took a longer time to degrade MWCNTs (multiple layers) with HRP compared to SWCNTs (one layer) [175]. HRP could not degrade double-walled CNTs, but MWCNTs could be partially degraded [176]. The degradation efficiency of CNTs is related to their own carboxylation degree, length, and oxidation degree [172]. The conformational changes in enzymes also play a key role in the process of removing NMs, such as in the removal of SWCNTs by MnP [177]. In addition, the functionalized modification of original SWCNTs also facilitated their degradation rate based on a strong interaction between SWCNTs and proteins [178]. After carboxyl modification, SWCNTs could interact electrostatically with Arg residues of the proteins [178]. The stability of the complexes produced by HRP and carboxylate CNT reactions was enhanced [175]. Due to the interaction between SWCNTs and proteins, the active sites of HRP were more accessible to the carboxylate SWCNT substrate [179]. Therefore, oxidative degradation of SWCNTs between the carboxyl groups of SWCNTs and positively charged structural regions of proteins is an effective method [180]. However, whether the biological activities of metabolites after these degradation processes are affected remains unclear.

## 7. Conclusions

The global NM market size was valued at USD 10.88 billion in 2022 and is expected to grow at a compound annual growth rate of 14.8% from 2023 to 2030 [181]. Bioactive NM supplies in the US market were estimated to be USD 130.17 billion before the end of 2021 [182]. The market size of NMs in microalgal bioproducts exploitation awaits further observation. Nevertheless, this review provides insights into the application prospects of nanotechnology to microalgae cultivation for improved high-value metabolite production. These metabolites include lipids, proteins, pigments, and EPSs. The NMs serve as new and supplemental solutions for the extant production of metabolites. In addition, NM treatment can lead to the structural and functional modification of microalgal metabolites, particularly polysaccharides. The main mechanisms by which NMs enhance algal biomass and metabolite production include improving photosynthetic utilization efficiency and regulating ROS levels. The removal of residual NMs in harvested metabolites has also gained much attention. Thus, three methods, including photodegradation, permanent magnet removal, and enzymatic degradation, have been developed. These methods will guarantee the safe use of NMs treatment-resulting bioproducts. In the future, precise elucidation of the mechanisms of NMs in the biosynthesis or functional alteration of metabolites, as well as the development of new functional NMs and relevant removal methods, are important research directions.

## Figures and Tables

**Table 1:** The effects of nanomaterials on the accumulation of microalgal metabolites.

Metabolites	NMs or NPs	Microalgal Species	Content or Change	Reference
Lipid	Fe	*Diacronema lutheri*	12 pg/cell	[104]
	ZnO	*Chlorella vulgaris*	~16% (saturated fatty acids) ~59% (polyunsaturated fatty acids)	[106]
	MgSO_4_		185.29 ± 4.53%	[110]
	Fe_2_O_3_ ZnO		13.8 wt% 14.25 wt%	[105]
	Cu Mg Pb Zn		0.28 ± 0.01 mg/L/day 0.46 ± 0.16 mg/L/day 0.65 ± 0.04 mg/L/day 0.74 ± 0.17 mg/L/day	[113]
	ZnO	*Halochlorella rubescens*	15~18%	[17]
	CNTs α-Fe_2_O_3_ MgO	*Tetradesmus obliquus*	14.65 ± 0.09 mg/L/day 18.77 ± 0.07 mg/L/day 15.94 ± 0.03 mg/L/day	[108]
	Fe	*Dunaliella tertiolecta*	35%	[109]
	Fe_3_O_4_	*Chlorella* sp. UJ-3	41.22 ± 1.62%	[11]
	MgAC-N(CD)s	*Tetraselmis* sp.	22.76%	[114]
	MgAC	*Haematococcus lacustris*	9446 pg/cell or 321 ± 2 mg/L	[122]
Total fatty acid	Fe_3_O_4_	*Chlorella* sp. UJ-3	235.33 ± 3.49 mg/g	[11]
Fatty acid methyl ester	C-paints	*Haematococcus lacustris*	1.6 × 10^−6^ mg/g	[121]
Astaxanthin	MgAC	*Haematococcus lacustris*	302 ± 69 pg/cell or 10.3 ± 0.4 mg/L	[131]
	Zn and Fe		7.29 mg/L	[129]
	ZnO		~20 mg/g DW	[18]
	N@CDs		66 mg/L	[135]
	C-paints		6.0 × 10^−8^ mg/cell	[121]
Carotene	BI_2_O_3_ Gd_2_O_3_ CdTe	*Dunaliella salina*	Approximately 120% Approximately 150% Approximately 110%	[117]
	Ag-Au	*Chlorella vulgaris*	Approximately 5 mg/L	[119]
β-carotene	MoS_2_ NPs	*Dunaliella salina*	0.15 mg/g	[118]
Phenolic compounds	TiO_2_	*Arthrospira platensis* *Haematococcus lacustris*	68.0 mg/g DW 65.2 mg/g DW	[138]
Exopolysaccharide	GO	*Nostoc flagelliforme*	46.4 mg/g DW	[12]

## Data Availability

The data presented in this review are available in the main text.
